# Evaluation of the Effectiveness and Accuracy of Non-Invasive Preimplantation Genetic Testing (niPGT) Compared to Invasive Embryo Biopsy

**DOI:** 10.3390/biomedicines13082010

**Published:** 2025-08-18

**Authors:** Charalampos Voros, Menelaos Darlas, Diamantis Athanasiou, Antonia Athanasiou, Aikaterini Athanasiou, Kyriakos Bananis, Georgios Papadimas, Charalampos Tsimpoukelis, Athanasios Gkirgkinoudis, Ioakeim Sapantzoglou, Ioannis Papapanagiotou, Dimitrios Vaitsis, Aristotelis-Marios Koulakmanidis, Vasileios Topalis, Nikolaos Thomakos, Marianna Theodora, Panagiotis Antsaklis, Fotios Chatzinikolaou, Hans Atli Dahl, Georgios Daskalakis, Dimitrios Loutradis

**Affiliations:** 1 1st Department of Obstetrics and Gynecology, ‘Alexandra’ General Hospital, National and Kapodistrian University of Athens, 80 Vasilissis Sofias Avenue, 11528 Athens, Greece; mdarlas2110@gmail.com (M.D.); tsimpoukelischa@gmail.com (C.T.); kimsap1990@hotmail.com (I.S.); aristoteliskoulak@gmail.com (A.-M.K.); thomakir@hotmail.com (N.T.); martheodr@gmail.com (M.T.); panosant@gmail.com (P.A.); gdaskalakis@yahoo.com (G.D.); 2 IVF Athens Reproduction Center, 15123 Maroussi, Greece; diamathan16@gmail.com (D.A.); antoathan16@gmail.com (A.A.); diamathan17@gmail.com (A.A.); 3 King’s College Hospitals NHS Foundation Trust, London SE5 9RS, UK; kyriakos.bananis@nhs.net; 4 Athens Medical School, National and Kapodistrian University of Athens, 15772 Athens, Greece; dr.georgepapadimas@gmail.com (G.P.); gpapamd@hotmail.com (I.P.); vaitsisdim@gmail.com (D.V.); vtopalismd@gmail.com (V.T.); loutradi@otenet.gr (D.L.); 5 Laboratory of Forensic Medicine and Toxicology, School of Medicine, Aristotle University of Thessaloniki, 54124 Thessaloniki, Greece; fotischatzin@auth.gr; 6 Amplexa Genetics A/S, 5000 Odense, Denmark; atli@amplexa.com; 7 Fertility Institute-Assisted Reproduction Unit, Paster 15, 11528 Athens, Greece

**Keywords:** non-invasive preimplantation genetic testing (niPGT), trophectoderm biopsy, embryonic cell-free DNA (cfDNA), in vitro fertilization (IVF), aneuploidy screening, next-generation sequencing (NGS), chromosomal mosaicism, blastocyst culture medium, embryo selection, assisted reproductive technology (ART)

## Abstract

**Background:** Preimplantation genetic testing for aneuploidy (PGT-A) is a popular approach in assisted reproductive technology that improves embryo selection and implantation rates. Traditional approaches rely on trophectoderm (TE) biopsy, which is an invasive procedure that might jeopardize embryo integrity and create technical constraints such as mosaicism-related misclassification. Non-invasive preimplantation genetic testing (niPGT) has emerged as a possible alternative, using embryonic cell-free DNA (cfDNA) extracted from wasted culture media or blastocoel fluid to assess chromosomal status without requiring direct embryo manipulation. **Methods:** This systematic study investigates the molecular mechanisms behind cfDNA release, its biological properties, and the technological concerns that influence its utilization in niPGT. We look at recent advances in next-generation sequencing (NGS), whole-genome amplification (WGA), and bioinformatic techniques that improve cfDNA-based aneuploidy detection. In addition, we compare the sensitivity, specificity, and concordance rates of niPGT to conventional TE biopsy, highlighting the major aspects impacting its diagnostic performance. **Results:** The release of cfDNA from embryos is influenced by apoptotic and necrotic processes, active DNA shedding, and extracellular vesicle secretion, which results in fragmented chromosomal material of different qualities and quantities. While niPGT has shown promise as a noninvasive screening approach, significant variability in cfDNA yield, maternal DNA contamination, and sequencing biases all have an impact on test accuracy. Studies show that niPGT and TE biopsies have moderate-to-high concordance, although there are still issues in detecting mosaicism, segmental aneuploidies, and DNA degradation artifacts. **Conclusions:** NiPGT is a safer and less intrusive alternative to TE biopsy, with potential clinical benefits. However, technical advancements are required to improve cfDNA collecting procedures, reduce contamination, and improve sequencing accuracy. Additional large-scale validation studies are needed to create standardized methodologies and ensure that niPGT achieves the diagnostic reliability requirements required for widespread clinical deployment in IVF programs.

## 1. Introduction

### 1.1. Background and Significance

PGT-A has transformed embryo selection in assisted reproductive technology (ART), enabling the identification of chromosomally normal embryos prior to implantation [1]. PGT-A aims to improve clinical pregnancy rates, minimize miscarriage rates, and increase the likelihood of a healthy live birth. Traditionally, PGT-A is conducted using a trophectoderm (TE) biopsy, in which a few cells from the blastocyst’s outer layer are collected and genetically examined [2]. While TE biopsy has considerably increased embryo selection accuracy, there are still worries about its invasive nature, the possibility of embryonic injury, and the danger of false-positive or false-negative outcomes due to embryonic mosaicism. According to the most recent ESHRE Good Practice Recommendations on Chromosomal Mosaicism, one of the most significant issues in PGT-A is detecting and interpreting chromosomal mosaicism, which complicates embryo selection and clinical decision-making. The guidelines emphasize mosaicism as a biological phenomena rather than a simply technical product, and they propose that embryos with low degrees of mosaicism may have developmental potential comparable to euploid embryos. This recommendation encourages further study into niPGT as a method of detecting chromosomal abnormalities without the potential bias induced by biopsy-related mosaicism misclassification [3]. These restrictions have prompted the creation of niPGT, which tries to assess cfDNA released by the embryo into the surrounding culture medium as an alternative method of genetic screening [4].

The potential of a biopsy-free, non-invasive alternative to traditional PGT-A has sparked widespread interest in reproductive medicine [5]. niPGT, which analyzes cfDNA in spent embryo culture medium or blastocoel fluid, provides a theoretically safer method for detecting aneuploidies [6]. However, before niPGT may replace TE biopsy in clinical practice, numerous fundamental issues must be solved. These include comprehending the molecular mechanisms that drive cfDNA release, calculating the proportion of cfDNA that appropriately represents the embryo’s chromosomal condition, and overcoming contamination from maternal or external DNA sources [7]. Furthermore, advancements in whole-genome amplification (WGA), NGS, and bioinformatics techniques are required to improve the accuracy of cfDNA-based diagnostics [8].

### 1.2. Cellular Pathways and Molecular Mechanisms of cfDNA Release

A variety of cellular mechanisms collaborate to regulate the release of cfDNA into embryo culture medium. These encompass apoptosis, necrosis, chromatin remodeling, and extracellular vesicle (EV) secretion. Each route possesses distinct molecular triggers and enzymatic mechanisms that influence the quantity, quality, and genomic representation of cfDNA. During apoptosis, caspase-3 and caspase-activated DNase (CAD/DFF40) cleave chromatin at internucleosomal regions, resulting in mono- and oligonucleosomal fragments typically measuring 50–200 base pairs in length. Upstream mitochondrial signaling and the formation of apoptosomes comprising cytochrome c, Apaf-1, and caspase-9 regulate the activation of these nucleases. During necrosis, the release of cfDNA is less regulated, resulting in DNA fragments of varying sizes and shapes. Moreover, modifications to histones during chromatin remodeling, including acetylation, methylation, and H3 citrullination by PAD enzymes, can facilitate nuclear condensation and DNA exposure. The chemical alterations, together with the continuous modifications in the cytoskeleton, render cfDNA distinct and influence its detectability in niPGT techniques.

The release of cfDNA into embryo culture media is a complicated process driven by several biological processes, each of which has a different impact on the quantity, quality, and composition of cfDNA [9]. Understanding these pathways is critical for improving niPGT and ensuring that identified cfDNA appropriately represents the embryo’s genetic state [10]. The chief sources of cfDNA include apoptotic DNA fragmentation, necrotic DNA release, active extracellular vesicle (EV)-mediated secretion, chromatin remodeling, and nuclear ejection [11]. Each of these routes has distinct molecular properties that affect the accuracy and interpretability of cfDNA-based embryo screening.

Apoptosis, also an active and controlled mechanism, is an important physiological mechanism during embryonic development that regulates cell turnover, eliminates faulty cells, and ensures appropriate cellular differentiation [12]. Apoptotic DNA release is distinguished by a well-organized nucleosomal fragmentation pattern, in which DNA is broken at precise internucleosomal areas by caspase-activated DNases [13]. This produces mono- and oligonucleosomal DNA fragments ranging in length from 50 to 200 base pairs (bp) [14].

The apoptotic cascade begins with the activation of caspases (e.g., caspase-3, caspase-7, and caspase-9), which causes chromatin condensation, DNA laddering, and nuclear fragmentation [12]. This fragmented DNA is then released into the extracellular environment via apoptotic bodies, which can rupture in culture medium and add to the cfDNA pool. Importantly, apoptotic cfDNA is low in molecular weight, degraded, and severely fragmented, differentiating it from necrotic or extracellular vesicle DNA [11].

One important concern in niPGT is whether apoptosis-derived cfDNA adequately represents the embryo’s chromosomal state. Apoptosis may exclusively impact genetically deficient or developmentally compromised blastomeres, indicating that the resultant cfDNA may exhibit a higher incidence of chromosomal abnormalities than the overall genomic composition of the viable embryo [15].

### 1.3. EV-Mediated DNA Secretion

One developing route for cfDNA release in preimplantation embryos is the secretion of DNA via EVs, including as exosomes and microvesicles [16]. Unlike apoptotic or necrotic DNA release, which occurs during passive cell-death processes, EV-mediated DNA secretion is thought to be an active and controlled mechanism [17]. This mechanism has been widely described in a variety of biological systems, including cancer biology and immune cell communication, and is now being studied in the context of early embryogenesis.

EVs are lipid bilayer-bound vesicles released by cells into the extracellular environment. They are essential for cell-to-cell communication, nucleic acid and protein transport, and microenvironmental control [18]. In embryo development, it is assumed that EVs promote communication between blastomeres and enable interactions with the maternal reproductive tract. Embryos release EVs into the culture medium, which include DNA, RNA, proteins, and signaling molecules that might reflect the embryo’s genetic and metabolic condition [19]. This type of DNA release differs fundamentally from that seen in apoptotic or necrotic cells, when DNA is released as a result of cellular breakdown. Instead, EV-associated DNA might be selectively packed and released, acting as a regulated mechanism for shedding genomic material [20].

The contents of EVs, including the DNA they transport, are assumed to be selectively loaded in response to the embryo’s developmental and metabolic condition [21]. Studies have demonstrated that DNA within EVs is frequently more stable than free cfDNA because the vesicular membrane shields it from enzymatic destruction [22]. This feature makes EV-derived cfDNA a potentially more accurate indicator for niPGT, since it may be a higher-quality source of DNA than apoptotic or necrotic cfDNA [23]. Furthermore, the DNA transported by EVs appears to be less fragmented than apoptosis-derived cfDNA, which is often broken down into small nucleosomal pieces. This shows that EV-derived DNA may give a more complete and undamaged representation of the embryonic genome, increasing the accuracy of genetic testing [24]. Although DNA transported by EVs may be less fragmented and more stable than cfDNA derived from apoptosis, it remains unclear whether DNA from EVs provides a comprehensive representation of the embryo’s entire chromosomal composition. Evidence suggests that extracellular vesicles (EVs) can transport extensive genomic regions; nevertheless, their packaging may render some loci more prevalent than the entire genome. Further research is required to ascertain the accuracy of EV-derived DNA in reflecting whole-chromosome status in niPGT [25].

Another important component of EV-mediated DNA secretion is its possible involvement in embryo-maternal signaling. It has been proposed that DNA-containing EVs might interact with maternal endometrial cells, altering implantation processes via modifying the maternal immunological response [26]. This idea raises important issues concerning cfDNA’s function in early pregnancy establishment, as well as whether embryos actively convey their genetic status to the maternal environment. If EV-mediated DNA release is a physiologically significant mechanism, it might shed fresh light on how embryos self-regulate and prepare for uterine implantation [16].

Despite its potential benefits, EV-derived cfDNA must overcome various obstacles before it can be employed successfully for niPGT. One of the primary challenges is separating EV-derived DNA from cfDNA generated during apoptosis or necrosis [27]. While EVs are expected to contain higher-quality DNA, the total cfDNA pool in embryo culture media is a combination of DNA from many sources. Current sequencing techniques do not distinguish between these separate populations, thus biasing the interpretation of niPGT data [28]. Furthermore, the quantity of DNA transported by EVs is modest, which may reduce the sensitivity of genetic analysis if cfDNA concentrations are inadequate for whole-genome sequencing [29].

Future advances in niPGT will necessitate the development of new methods for extracting and analyzing EV-derived DNA, as well as the refinement of sequencing technology to discriminate between distinct cfDNA subtypes [30]. Ultracentrifugation, size-exclusion chromatography, and immunoaffinity capture techniques may improve EV selective separation, letting researchers study their DNA composition with more precision. Furthermore, developments in single-molecule sequencing and methylation profiling may aid in distinguishing EV-associated cfDNA from apoptotic or necrotic cfDNA, increasing the accuracy of embryo genetic evaluations.

### 1.4. Chromatin Remodeling and Nuclear Expulsion Events

Embryonic development is characterized by significant chromatin remodeling, a dynamic process that is critical for gene control, DNA replication, and cellular differentiation. Chromatin remodeling involves structural changes to the DNA–protein complex that enable correct transcriptional activation, repression, and genome organization [31]. During preimplantation development, embryonic cells undergo considerable nuclear remodeling, which may lead to the release of cfDNA into the culture medium [16]. While much of the cfDNA found in wasted media has been ascribed to apoptotic and necrotic processes, there is growing evidence that part of this extracellular DNA is derived from active nuclear remodeling and chromatin ejection mechanisms [32].

One of the most important processes in early embryogenesis is the maternal-to-zygotic transition (MZT), which occurs when control of gene expression is switched from maternal transcripts to the newly activated embryonic genome [33]. This change is followed by extensive chromatin remodeling, histone alterations, and epigenetic reprogramming, which allow the embryo to build a distinct gene-expression profile required for continuing development [34]. During this process, unneeded or damaged DNA segments may be selectively degraded and ejected from the nucleus, therefore adding to the cfDNA pool [35]. The elimination of unnecessary DNA sequences is crucial for preserving genomic integrity and minimizing transcriptional interference from redundant genetic components.

As the embryo develops through cleavage divisions and compaction, nuclear architecture reorganizes to facilitate efficient DNA replication and chromatin accessibility. This remodeling process includes nucleosome relocation, DNA demethylation, and histone changes, which together influence the embryo’s developmental trajectory [36]. In rare situations, chromatin fragments that are no longer needed may be actively destroyed and expelled from the nucleus, resulting in the presence of cfDNA in the extracellular environment. Compared to cfDNA formed from apoptosis or necrosis, chromatin remodeling-related cfDNA may be a more controlled and physiologically relevant source of extracellular DNA, possibly providing vital insights into embryonic development [37].

In addition to chromatin rearrangement, nuclear ejection of micronuclei, which are tiny extranuclear entities containing missegregated chromosomes or DNA fragments, might contribute to cfDNA release. Micronuclei development is a well-known event in cells experiencing chromosomal stress, replication mistakes, or DNA damage repair [38]. These micronuclei can either be reintegrated into the main nucleus or removed from the cell via exocytosis, adding to the cfDNA pool [39]. If comparable ejection mechanisms exist in early embryos, this might explain why cfDNA content in culture medium varies between embryos and why some embryos produce higher-quality cfDNA than others.

Another fascinating theory is that nuclear ejection mechanisms work as a genomic quality control system, allowing embryos to actively remove defective or unneeded DNA. This procedure may be especially crucial in the removal of transposable elements, repetitive sequences, and altered DNA regions that might jeopardize genomic integrity [40]. The selective elimination of such components would help to create a more stable and transcriptionally competent embryonic genome, improving the chances of successful implantation and development.

From the standpoint of niPGT, the presence of cfDNA resulting from chromatin remodeling and nuclear ejection presents both obstacles and opportunities. On the one hand, if cfDNA contains a considerable amount of extruded nuclear material, it may provide a more accurate representation of the embryo’s real chromosomal makeup than apoptosis- or necrosis-derived DNA [9]. However, if ejected chromatin is largely composed of genomically unstable or selectively degraded areas, it may not accurately reflect the genetic condition of viable embryonic cells. This raises crucial issues about how cfDNA content varies throughout preimplantation development and whether specific phases of chromatin remodeling correlate with enhanced diagnostic accuracy in niPGT [41].

## 2. Material and Methods

### 2.1. Study Design and Systematic Review Protocol

This systematic study followed the Preferred Reporting Items for Systematic Reviews and Meta-Analyses (PRISMA) standards to guarantee methodological rigor, transparency, and reproducibility when evaluating niPGT. The study protocol was pre-registered in the PROSPERO database (CRD420250654550) to improve methodological reliability and guarantee that standard practices in reproductive medicine systematic reviews are followed. The major goal of this study was to evaluate the diagnostic accuracy, clinical value, and technical advances of niPGT in comparison to TE biopsy-based PGT-A. This study also aims to investigate the biological and technological variables impacting cfDNA-based aneuploidy diagnosis and make evidence-based suggestions for its prospective inclusion into ordinary clinical practice.

The purpose for this comprehensive analysis derives from the growing interest in non-invasive genetic testing approaches that avoid the hazards associated with TE biopsy. While TE biopsy has considerably improved embryo selection in IVF cycles, concerns about embryo survival, possible biopsy-induced mosaicism misclassification, and procedural variability have led to the search for alternatives such as niPGT. Since cfDNA released into embryo culture media has been found as a promising source of embryonic genetic material, it is critical to assess the diagnostic validity and practicality of niPGT in a clinical environment. This study uses a comprehensive synthesis of the current research to assess if niPGT is a reliable and effective alternative to invasive biopsy-based procedures.

An organized and thorough search approach was devised to discover peer-reviewed articles on cfDNA-based aneuploidy screening in human preimplantation embryos. Studies were considered if they reported quantifiable values of sensitivity, specificity, concordance rates, or clinical outcomes for niPGT and TE biopsies. The primary research questions addressed in this review are how niPGT compares to TE biopsy in terms of accuracy, what factors contribute to variability in cfDNA collection, sequencing, and analysis, and how embryo developmental stage, culture medium composition, and maternal DNA contamination affect cfDNA yield and test reliability. Furthermore, this study investigates the constraints that now hinder niPGT from being extensively utilized in clinical practice and provides alternative ways to address these issues.

To ensure the inclusion of high-quality research, rigorous eligibility criteria were used, with an emphasis on methodological rigor, adequate sample size, and validation against TE biopsy as the reference standard. In addition to assessing cfDNA fragmentation patterns, sequencing depth, and amplification efficiency, this study investigated how bioinformatics processing, detection thresholds, and mosaicism interpretation affect niPGT outcomes. The ESHRE Good Practice Recommendations on Chromosomal Mosaicism underline the importance of consistent reporting criteria in embryo selection, particularly for mosaic embryos. These standards categorize chromosomal mosaicism based on aneuploid cell proportion: ≤20% euploid, ≥80% aneuploid, and intermediate levels as mosaic. However, niPGT provides distinct hurdles in mosaicism identification, owing to cfDNA fragmentation, sequencing biases, and probable maternal DNA contamination. Given these constraints, ESHRE recommends that laboratories develop established criteria for evaluating mosaicism in cfDNA-based PGT to ensure accuracy and consistency in clinical reporting [3]. This review attempted to identify potential causes of bias and heterogeneity across different research, which might affect the repeatability and accuracy of niPGT findings, by critically examining these characteristics.

Given the methodological variances in niPGT methods, this review investigated variations in cfDNA-extraction techniques, sequencing technology, embryo culture conditions, and analytical processes. Identifying and resolving these discrepancies is critical for future standardization of niPGT methods and ensuring comparability among clinical laboratories. Furthermore, this systematic review identifies gaps in the available literature and suggests future research initiatives to improve the sensitivity and specificity of niPGT. By synthesizing the most recent findings, this review seeks to give scientifically supported suggestions for improving niPGT techniques and expanding their practicality for wider clinical implementation in IVF programs.

### 2.2. Search Strategies and Literature Selection

A complete search was undertaken throughout PubMed, Embase, Web of Science, Scopus, and the Cochrane Library, encompassing all relevant research published up to the current day. The search approach was developed to optimize sensitivity while retaining specificity by combining MeSH phrases with free-text keywords relevant to preimplantation genetic testing, cfDNA analysis, and chromosomal aneuploidy detection. To make sure that every relevant study was taken into account, search phrases had to include “niPGT,” “cfDNA,” “extracellular DNA,” “spent culture medium,” “blastocoel fluid,” “PGT-A,” “trophectoderm biopsy,” “aneuploidy screening,” “chromosomal abnormalities,” “NGS,” plus “WGA.”

Following the first search, duplicate entries were deleted using automatic filtering, followed by manual verification to verify correctness. The remaining papers were screened for titles and abstracts to exclude irrelevant research, followed by full-text reviews to determine eligibility using predetermined inclusion and exclusion criteria. Additional papers were discovered after manually searching reference lists from significant journals and pertinent reviews in the area.

### 2.3. Eligibility Criteria

This systematic review included studies that directly compared niPGT to TE biopsy-based PGT-A in human IVF cycles, reported diagnostic accuracy measures such as sensitivity, specificity, PPV, and NPV, and investigated cfDNA yield, fragmentation profiles, or molecular mechanisms underlying cfDNA release. Furthermore, only research that used proven molecular methods like NGS, WGA, or quantitative PCR were included. Inclusion criteria were clinical trials, retrospective cohort studies, case–control studies, and observational studies with well-defined methodology. Exclusion criteria included research using animal models instead of human embryos, review papers, conference abstracts, comments, and studies with no original data. Research was also rejected if it provided insufficient or incomplete data on the comparison of niPGT and TE biopsies, or if it relied exclusively on exploratory studies without strong clinical validation.

### 2.4. Data Extraction and Quality Assessment

Two impartial reviewers carefully extracted data using a standardized data-collecting form. The extracted data includes study information like author, year, sample size, and study type, as well as technical procedures including cfDNA source, sequencing platform, and analytic techniques. Diagnostic performance measures like sensitivity, specificity, concordance rates, and false-positive/false-negative rates were measured. Furthermore, technical aspects impacting cfDNA detection were evaluated, including cfDNA yield, fragmentation index, detection limits, and maternal DNA contamination levels. When available, clinical outcomes such as implantation rate, pregnancy rate, and live birth rate were documented.

The included studies’ methodological quality was evaluated using the QUADAS-2 (Quality Assessment of Diagnostic Accuracy Studies—2) methodology, which assesses the risk of bias and applicability issues. The studies were reviewed for patient selection bias, index test reliability (niPGT technique), reference standard validity (TE biopsy concordance), and flow/timing issues for cfDNA sample and sequencing methods. Studies with a high risk of bias or ambiguous reporting were identified, and sensitivity analyses were carried out to determine the influence of study quality on overall findings.

The table below summarizes the quality assessment of the included studies using the QUADAS-2 criteria. This assessment looked at the potential of bias in research design, the dependability of niPGT techniques, and the general relevance of the findings.

Subgroup studies were undertaken to look into potential factors impacting niPGT diagnostic performance. Studies were classified according to cfDNA source (spent culture media vs. blastocoel fluid), embryo developmental stage (Day 3 cleavage stage vs. Day 5/6 blastocysts), and sequencing methods (next-generation sequencing, whole-genome amplification, or qPCR-based methodologies). The impact of culture medium composition on cfDNA release and stability was also investigated, as variations in media formulations and embryo metabolism might affect DNA fragmentation patterns. To assess the robustness of the findings, sensitivity analyses were conducted, omitting studies with limited sample numbers, high degrees of selection bias, or non-standardized cfDNA processing techniques.

### 2.5. Quality Assessment of Included Studies

Table 1 shows the quality evaluation of research comparing niPGT to TE biopsy-based PGT-A, using NOS. The study’s qualities, methodological rigor, and danger of bias are assessed. The Author/Year column contains the principal author’s name and the year of publication. Cohort studies, observational studies, retrospective analyses, pilot studies, and multicenter trials are among the research designs classified as Study Type. Cases (Number) denotes the sample size, which reflects statistical power and generalizability. Groups identify research comparisons, with all studies comparing niPGT-A to TE biopsy-based PGT-A. Interventions discuss cfDNA-collecting methods (spent culture media or blastocoel fluid) and sequencing techniques (NGS, WGA, qPCR). Selection Bias evaluates participant representativeness and inclusion criteria. Comparability assesses how successfully research adjusted for confounding factors such mother’s age, embryo quality, and culture conditions. Outcome Assessment assesses diagnostic accuracy, clinical pregnancy rates, embryo implantation success, and consistency with TE biopsy results. The Total NOS Score (0–9) measures overall study quality, with higher values suggesting stronger methodology. The risk of bias is classified as low, moderate, or high according to methodological strengths and limits. This table presents an objective assessment of niPGT dependability in clinical practice, indicating methodological discrepancies and potential topics for future study standardization.

Table 1 indicates the quality assessment of the included studies reveals substantial methodological diversity in the assessment of niPGT against TE biopsy-based PGT-A. Differences in research design, sample size, intervention techniques, selection bias, comparability, and outcome evaluation all have an influence on the findings’ dependability and repeatability. These differences must be carefully examined to determine the benefits and limits of niPGT as a clinical alternative to TE biopsy.

The included papers are a combination of cohort studies, observational research, retrospective analysis, pilot studies, and multicenter trials, demonstrating the changing nature of niPGT research. Cohort studies, such as those conducted by Rubio et al. (2020) and Chen et al. (2025), offer prospective, longitudinal data on embryo selection results, implantation rates, and live birth success [42,49]. These studies often give higher-quality data than retrospective analyses because they allow for greater control of confounding factors and more solid clinical insights [42,49]. However, observational and retrospective investigations, such as those conducted by Lledo et al. (2021) and Yin et al. (2021), may introduce selection bias and lack uniformity in embryo assessment, potentially resulting in variations in diagnostic accuracy between niPGT and TE biopsies [44,47].

Kuznyetsov et al. (2018) conducted pilot experiments to assess technical feasibility, including cfDNA production, sequencing depth, and concordance rates with TE biopsy [46]. While these studies provide valuable early data, their limited sample sizes restrict their statistical power and applicability to clinical situations [46]. Larger multicenter studies, such as Sialakouma et al. (2021), provide more robust results by combining data from several IVF clinics, minimizing institutional biases and enhancing external validity [23]. However, multicenter studies include diversity in laboratory circumstances, sequencing processes, and embryo-handling approaches, which may impact repeatability [23].

The sample sizes in these research vary from 11 embryos (Kulmann et al., 2021) to 484 embryos (Rubio et al., 2020) [42,52]. Larger sample sizes improve statistical power and reliability, lowering the impact of random variability and increasing confidence in reported findings. Studies with a larger number of cases, such as Rubio et al. (2020), Sialakouma et al. (2021), and Chen et al. (2025), are more likely to reach solid conclusions on the diagnostic accuracy of niPGT [23,42,49]. In contrast, studies with lower sample numbers, such as Yeung et al. (2019) and Chen et al. (2020), may be more subject to variations in cfDNA yield, sequencing artifacts, and selection bias, which may affect their stated concordance rates with TE biopsy [50,51]. The variety in sample sizes among research emphasizes the need for bigger, well-powered, prospective clinical trials to validate niPGT and prove its repeatability in various laboratory and clinical contexts. The inclusion of smaller studies in this dataset indicates that niPGT research is still in the exploratory stage, requiring additional refining before clinical use.

Selection bias is an important issue in determining the credibility of study results. Table 1 displays selection bias ratings ranging from 2/4 to 3/4, demonstrating substantial heterogeneity in research inclusion criteria. Studies with stronger selection-bias control scores, such as Rubio et al. (2020), employed unambiguous, well-defined embryo selection processes and strict inclusion/exclusion criteria [42]. These trials are more trustworthy in measuring the efficacy of niPGT because they reduce variability caused by changes in embryo quality, mother’s age, and ovarian stimulation methods [42]. In contrast, studies with lower selection-bias ratings, such as Kuznyetsov et al. (2016) and Kulmann et al. (2021), may have added inconsistency in patient selection, leading to uneven cfDNA yields and varying concordance rates with TE biopsy [46,52]. The occurrence of selection bias in certain studies may explain some of the observed differences in niPGT diagnostic accuracy, indicating that standardization of inclusion criteria is required for future study [46,52].

Study comparability ratings were between 1/2 and 2/2, indicating how well confounding variables were controlled. Studies scoring 2/2, such as Sialakouma et al. (2021), controlled for significant variables such maternal age, embryo culture circumstances, and blastocyst quality [23]. These characteristics are important in niPGT research since cfDNA release and fragmentation might vary depending on embryo developmental stage, chromosomal mosaicism, and metabolic activity [23]. Studies with lower comparability ratings, such as Yeung et al. (2019) and Sun et al. (2023), may not have sufficiently controlled for confounding factors, resulting in heterogeneity in cfDNA quality and sequencing performance [48,51].

The outcome evaluation ratings ranged from 2/3 to 3/3, indicating variations in how niPGT data were verified versus TE biopsy findings. High-scoring studies, like Rubio et al. (2020), Lledo et al. (2021), and Huang et al. (2022), used well-defined outcome measures, in-depth sequencing methods, and robust statistical analyses [42,43,44]. These studies offered better evidence supporting niPGT accuracy, since their methodology allowed for rigorous comparisons of cfDNA-based and TE biopsy-based aneuploidy identification [42,43,44]. However, studies with lower outcome-evaluation ratings, such as Kuznyetsov et al. (2016) and Kulmann et al. (2021), showed higher variability in their results, which might be attributable to variable cfDNA-collecting procedures, sequencing depth, or embryo selection criteria [46,52]. Variability was also influenced by differences in cfDNA collection, whether from spent culture medium (SCM) or blastocoel fluid (BF) in concordance rates between niPGT and TE biopsy.

The overall NOS ratings varied from 5/9 to 8/9, with research categorized as low, moderate, or high risk of bias. Low-risk studies included Rubio et al. (2020), Huang et al. (2022), and Chen et al. (2025), all of which displayed good methodological rigor, explicit embryo selection criteria, and robust sequencing techniques [42,43,49]. These studies give the most credible evidence of niPGT accuracy and clinical applicability [42,43,49]. Mild-risk studies, like Lledo et al. (2021) and Yin et al. (2021), revealed certain limitations, such as lower sample numbers, mild selection bias, or minor methodological errors [44,47]. While these findings add to the expanding data basis for niPGT, they need to be validated in bigger, more standardized investigations [44,47]. High-risk studies, such as Kulmann et al. (2021) and Kuznyetsov et al. (2016), have severe methodological flaws, including limited sample numbers, inconsistent cfDNA-extraction procedures, and insufficient control of confounding factors [46,52]. These studies should be regarded with caution since the results may not be replicated in bigger, well-controlled trials [46,52].

The retrieved data were analyzed qualitatively, with findings compared across studies to determine trends in niPGT diagnostic accuracy, concordance rates with TE biopsy, and clinical outcomes. To account for any heterogeneity across trials, a random-effects meta-analysis was performed when needed. Heterogeneity was assessed using the I^2^ statistic and Cochran’s Q test, with thresholds of 25%, 50%, and 75% indicating low, moderate, and high heterogeneity, respectively. Sensitivity analyses were carried out by omitting studies with a high probability of bias to assess their influence on overall findings. Furthermore, publication bias was evaluated using Egger’s and Begg’s tests, and possible bias was shown using funnel plots where a significant number of papers were available. In addition to evaluating study quality with the QUADAS-2 (Quality Assessment of Diagnostic Accuracy Studies—2) tool, the overall certainty of evidence was assessed using the GRADE (Grading of Recommendations, Assessment, Development, and Evaluation) framework. This technique assessed evidence strength as high, moderate, low, or very low based on variables such as bias risk, study inconsistency, indirectness, imprecision, and publication bias. Any differences in quality rating between reviewers were handled through discussion or arbitration by a third reviewer, ensuring a consistent and unbiased evaluation of the included studies.

All research included in this systematic review used human embryos and were required to have Institutional Review Board (IRB) permission from their respective institutions. Ethical issues were examined in accordance with globally known research ethics norms, such as the Declaration of Helsinki and Good Clinical Practice (GCP) standards. Studies were only included if they expressly stated that they had informed permission from embryo donors, assuring conformity with ethical guidelines in reproductive research. The possible implications of niPGT for clinical decision-making were discussed in light of responsible embryo selection methods, underlining the importance of continued ethical conversation in the area.

### 2.6. cfDNA Collection, Processing, and Sequencing Analysis

The variability in cfDNA-collection methodologies, sequencing platforms, and bioinformatics workflows has a significant impact on cfDNA yield, maternal DNA contamination, and overall diagnostic reliability. Table 2 provides a comparative summary of these molecular and technical parameters across the included studies, highlighting key challenges and variations in niPGT.

The heterogeneity in cfDNA collection, sequencing procedures, and bioinformatics approaches has a major impact on the accuracy, reliability, and clinical usefulness of niPGT. Table 2 compares these molecular and technological parameters, showing how different techniques affect cfDNA integrity, sequencing efficiency, and aneuploidy detection rates.

A significant problem in niPGT is the poor yield and considerable fragmentation of cfDNA, which varies according to the collecting technique. Studies utilizing SCM as a cfDNA source, such as Rubio et al. (2020) and Huang et al. (2022), found moderate-to-low yields, which were frequently accompanied by substantial maternal DNA contamination [42,43]. This problem occurs because maternal DNA from granulosa and cumulus cells might enter the culture media during oocyte extraction and fertilization, resulting in false-positive aneuploidy findings [42,43]. Studies using BF, such as Kuznyetsov et al. (2016), found lower levels of maternal contamination, indicating that BF may be a more pure source of embryonic cfDNA [46]. However, BF aspiration needs micromanipulation, making it slightly invasive, and this might raise concerns regarding its classification as a truly non-invasive technique [46].

Another aspect influencing niPGT accuracy is the sequencing platform employed. Most investigations used NGS, which allows for in-depth sequencing and identification of complicated chromosomal abnormalities such as segmental deletions and duplications. However, variations in bioinformatics processes, such as CNV detection, SNP filtering, and mosaicism evaluation, lead to diversity in diagnostic performance. For example, Xu et al. (2016) and Kulmann et al. (2021) found significant false-positive/negative rates due to constraints in cfDNA read-depth coverage, implying that variations in bioinformatics analysis may lead to disparities in aneuploidy detection [52,53].

Maternal DNA contamination is still one of the most severe limitations of niPGT. Studies by Yin et al. (2021) and Sialakouma et al. (2021) found moderate quantities of maternal DNA in cfDNA samples, indicating that bioinformatics filtering methods must be improved to distinguish embryonic cfDNA from maternal contamination [23,47]. Some new approaches use epigenetic and methylation-based filtering techniques to discriminate between embryo-derived and maternal cfDNA, although these methods require additional confirmation. False-positive and false-negative rates are still a major problem for niPGT dependability [23,47]. Studies by Xu et al. (2016) and Kulmann et al. (2021) found substantial false-negative rates, suggesting that certain aneuploid embryos may emit disproportionately low quantities of cfDNA, leading to euploid diagnosis [52,53]. Rubio et al. (2020) found moderate false-positive rates, indicating that some euploid embryos were incorrectly categorized as aneuploid due to technical errors in cfDNA sequencing [42]. These findings highlight the need of standardizing cfDNA extraction, setting quality-control limits, and improving bioinformatics pipelines to ensure the accuracy of niPGT-based embryo selection.

Despite these restrictions, Table 2 shows numerous advancements in cfDNA-based aneuploidy detection. The growing usage of NGS-based niPGT, as demonstrated by studies such as Sialakouma et al. (2021) and Chen et al. (2025), has increased diagnostic sensitivity and specificity, notably for detecting whole-chromosome aneuploidies [23,49]. However, segmental anomalies and mosaicism identification remain difficult, necessitating more study into deep sequencing, machine learning-based classification, and multi-omics integration to improve niPGT performance. Finally, the findings reported in Table 2 highlight the importance of standardizing cfDNA-collection techniques, sequencing technologies, and bioinformatics tools in order to increase niPGT diagnosis accuracy. Future study should concentrate on increasing cfDNA output, reducing maternal DNA contamination, and improving bioinformatics analysis to improve the reliability of non-invasive embryo selection in ART.

## 3. Results

### 3.1. Study Selection and PRISMA Flow Diagram

**Scheme 1:**
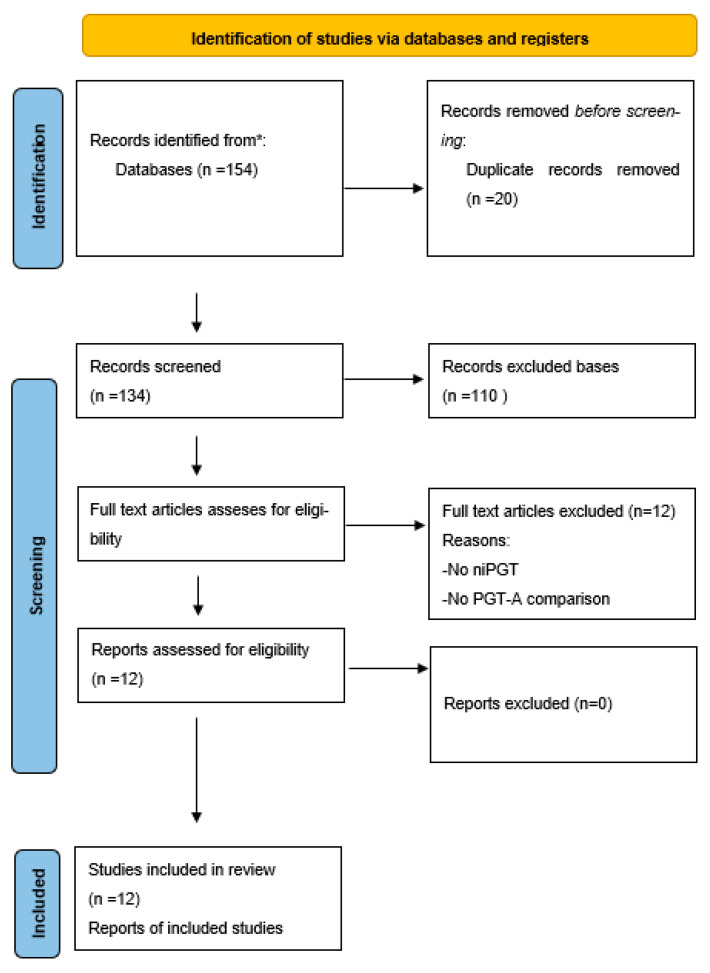
PRISMA flow diagram for study selection process. * Consider, for example, databases, registers, websites, organisations, reference lists.

The PRISMA (Preferred Reporting Items for Systematic Reviews and Meta-Analyses) flow diagram in Scheme 1 depicts the systematic method of identifying, screening, determining eligibility, and included studies in this review [54]. The identification step started with the retrieval of 154 records from electronic databases, which was followed by the elimination of 20 duplicate records to guarantee that each study was tallied once. After the duplicates were deleted, the remaining 134 records went through the screening step, where their titles and abstracts were evaluated. At this point, 110 records were removed because they were irrelevant to the study objective, including studies that did not focus on niPGT or did not provide a direct comparison to PGT-A by trophectoderm biopsy. During the eligibility phase, 24 full-text papers were reviewed. Twelve of these were eliminated for specific reasons, such as the absence of niPGT-related data, the lack of a comparison group using PGT-A, publishing in a language other than English, or methodological errors that did not match inclusion requirements. The final inclusion phase produced 12 papers that matched all eligibility criteria and were included in the systematic review. These studies serve as the foundation for assessing the accuracy, clinical value, and methodological advances of niPGT in comparison to TE biopsy-based PGT-A.

This flow diagram follows the PRISMA 2020 principles, promoting openness in research selection while reducing selection bias. This methodical technique improves repeatability and dependability by documenting each stage of the inclusion process [54].

The PRISMA flow diagram, in Scheme 1, provides an important visual depiction of the research selection process, providing methodological transparency and repeatability in this systematic review. It clearly shows how studies were located, screened, evaluated for eligibility, and eventually included in the final analysis. This organized method is critical for reducing selection bias, documenting the criteria used to reject research, and ensuring consistency in evidence synthesis. The huge number of studies originally obtained (154 entries from electronic databases) was an important finding during the identification phase. The elimination of 20 duplicate items emphasizes the need of eliminating data redundancy, especially in systematic reviews that use various databases and may be indexed numerous times. Including each study only once eliminates duplicate counting and ensures the dataset’s integrity. The following title- and abstract-screening process reduced the selection to 134 distinct papers, indicating a critical filtering step in finding the most pertinent literature.

The screening process is crucial for removing research that do not match the fundamental inclusion requirements. Here, 110 studies were removed, resulting in a significant decrease in the dataset. This exclusion rate indicates that a sizable fraction of the initially retrieved papers were either not directly relevant to niPGT, did not include a comparison with TE biopsy-based PGT-A, or lacked main research data. The large number of exclusions at this stage emphasizes the need of a well-defined and specific search strategy, which ensures that only papers that directly address the study objectives advance to full-text review.

The eligibility step included a thorough examination of the remaining 24 papers, as well as a full-text assessment to ensure that they satisfied all methodological and reporting standards. At this point, 12 further studies were removed, representing a sizable fraction of the full-text evaluated papers. The exclusion criteria in the PRISMA diagram highlight numerous prevalent concerns that restrict the relevance of specific studies to niPGT research. These include a paucity of niPGT-related data, the absence of a direct PGT-A comparison, publications in languages other than English, and methodological errors. Language exclusions indicate that research published in languages other than English were excluded owing to translation constraints or methodological consistency problems. The elimination of papers due to methodological discrepancies shows that some studies lacked consistent methods, rigorous statistical analyses, or clearly stated outcome measures, thereby introducing bias into the evaluation.

The final inclusion phase yielded 12 studies that met all eligibility requirements. This figure offers a modest yet extremely useful dataset for systematic study. The careful selection of studies keeps the evidence base narrow, methodologically sound, and relevant to the study issue. The studies included in this systematic review will help to evaluate the accuracy, efficacy, and clinical utility of niPGT versus TE biopsy-based PGT-A, laying the groundwork for future analyses of diagnostic performance, cfDNA collection methods, and sequencing technologies. Some systematic reviews use conference abstracts, clinical trial registries, or preprints to reduce publication bias, which arises when studies with poor or equivocal outcomes are underreported. While excluding gray literature is not always harmful, it should be justified in the Section 2 (Materials and Methods) to promote clarity in research selection criteria. Furthermore, the removal of non-English research may add linguistic bias, thereby restricting the generalizability of the findings if pertinent studies in other languages were omitted.

The PRISMA flow diagram efficiently chronicles the systematic review process, exhibiting thorough study screening, unambiguous exclusion criteria, and a methodologically sound selection procedure. However, to improve the review, it would be helpful to specifically explain how inter-reviewer agreement was determined throughout screening and full-text selection. Using criteria such as Cohen’s kappa statistic would give an objective measure of consistency across reviewers, ensuring that research selection decisions were not influenced by personal bias. Furthermore, a sensitivity study might investigate if incorporating papers removed due to methodological discrepancies will materially affect the review’s results.

### 3.2. Summery of Studies and Primary Outcomes

#### 3.2.1. Study Design and Methodological Considerations: Assessing the Strengths and Weaknesses of Each Approach

Table 3 shows that heterogeneity in research design has a considerable influence on the trustworthiness of reported findings. The collection includes cohort studies, observational research, retrospective analyses, pilot studies, and multicenter trials, each with a varying level of evidence quality and statistical rigor. Cohort studies, including Rubio et al. (2020), Chen et al. (2025), and Huang et al. (2022), provide prospective, longitudinal data on embryo development and clinical outcomes following niPGT-based embryo selection [42,43,50]. These studies give strong clinical evidence by reducing recollection bias and enabling controlled data gathering [42,43,50]. However, prospective studies need a longer follow-up timeframes as well as significant financial and logistical resources, which may restrict sample numbers and generalizability. Retrospective investigations, such as Lledo et al. (2021), Yin et al. (2021), and Kulmann et al. (2021), rely on previously gathered clinical data, allowing for a speedier study of niPGT performance in real-world situations [44,47,52]. However, selection bias and differences in cfDNA-handling techniques between laboratories may cause variances in niPGT and TE biopsy concordance rates. The dependence on stored SCM samples may potentially cause cfDNA degradation, reducing sequencing accuracy.

Observational studies, such as Xu et al. (2016), provide technical validation for cfDNA-based aneuploidy screening, with an emphasis on cfDNA stability, fragmentation patterns, and sequencing accuracy [53]. These investigations are critical for boosting WGA efficiency, increasing NGS coverage, and fine-tuning bioinformatics workflows for maternal DNA filtering [53]. Pilot studies, such as Kuznyetsov et al. (2016), investigate the possibility of collecting and sequencing cfDNA from blastocoel fluid [46]. These studies give proof-of-concept evidence, but the tiny sample numbers restrict statistical significance [46]. Furthermore, micropipette suction of blastocoel fluid is a minimally invasive approach that some researchers believe may still alter embryonic growth and implantation potential. Multicenter studies, like Sialakouma et al. (2021), use data from different IVF clinics to reduce institutional bias and increase external validity [23]. However, inter-laboratory discrepancies in cfDN- extraction techniques, sequencing depth, and embryo culture conditions make direct comparisons of niPGT accuracy across clinical contexts challenging. The absence of RCTs in Table 3 is a significant drawback, as RCTs give the greatest degree of clinical evidence by reducing confounding variables [23].

#### 3.2.2. Sample Size and Statistical Power: Impact on Diagnostic Performance and Clinical Generalizability

Sample size has a direct impact on the statistical power of niPGT research, altering the reliability of sensitivity, specificity, and concordance rate estimations. Table 3 shows sample sizes ranging from 11 embryos (Kulmann et al., 2021) to 484 embryos (Rubio et al., 2020), resulting in differences in research robustness [42,52]. Larger sample numbers, such as those used by Rubio et al. (2020) and Sialakouma et al. (2021), provide more confidence in diagnostic accuracy estimates, lowering the chance of random changes in cfDNA sequencing findings [23,42]. These investigations are more adapted to finding low-frequency genetic anomalies such segmental deletions, duplications, and chromosomal mosaicism [23,42]. In contrast, small-scale research, such as Yeung et al. (2019) and Kulmann et al. (2021), may overestimate or underestimate false-positive and false-negative rates, resulting in reduced repeatability [51,52]. These studies frequently lack statistical power to confirm uncommon embryonic aneuploidies, making their results less applicable to larger IVF populations [51,52]. Interestingly, several of the studies in Table 3 do not explicitly disclose power estimates, which are critical for establishing if their sample sizes were sufficient to detect statistically significant differences in niPGT vs. TE biopsy results. Future research should use power assessments to guarantee that the stated findings are not due to underpowered study designs.

#### 3.2.3. Variability in cfDNA-Collection Methods: Implications for DNA Integrity and Sequencing Accuracy

The method for collecting cfDNA is one of the most important variables impacting the accuracy of niPGT, since it has a direct influence on DNA integrity, sequencing reliability, and the capacity to identify chromosomal abnormalities. Table 3 shows the variety in cfDNA-collecting strategies across different research, reflecting the ongoing debate over which methodology produces the best representative and high-quality embryonic DNA for aneuploidy screening. The three most prevalent sources of cfDNA in niPGT research are SCM, blastocoel fluid (BF), and blastocyst culture media, each with unique advantages and disadvantages.

The majority of research, including those by Rubio et al. (2020), Lledo et al. (2021), and Kulmann et al. (2021), utilized cfDNA isolated from wasted culture media [42,44,52]. This approach is completely noninvasive, making it the most appealing alternative for clinical translation. Spent culture media is normally enriched in cfDNA fragments produced by embryos when developing in vitro [42,44,52]. However, various molecular obstacles restrict its efficacy. One key challenge is the low concentration of embryonic cfDNA in SCM, making it difficult to collect enough DNA for accurate sequencing. Because cfDNA is passively released into the culture media, the amount collected varies greatly between embryos, resulting in variable detection rates for aneuploidies. Furthermore, SCM cfDNA is extremely fragmented, with studies indicating that the majority of DNA pieces are fewer than 150 base pairs long, making reliable WGA and next-generation sequencing difficult.

Another key difficulty with cfDNA obtained from wasted culture media is maternal DNA contamination. During oocyte retrieval and fertilization, granulosa and cumulus cells around the oocyte may be added to the embryo culture plate [55]. These maternal cells disintegrate during time, releasing cfDNA that can combine with embryonic cfDNA in the culture media. The level of maternal DNA contamination varies between research; certain investigations have identified elevated levels of maternal DNA signal, particularly in SCM collected during the early cleavage phases (Day 3 of embryonic development). The level of contamination appears to be contingent upon the handling of embryos, the culture system employed, and the cfDNA isolation techniques utilized [27]. This contamination skews niPGT results because it may falsely reflect aneuploidies that do not exist in the embryo. To address this issue, researchers have tried using epigenetic markers, SNP profiling, and bioinformatics filtering approaches to differentiate between maternal and embryonic cfDNA. However, no standardized procedure is currently available, and the efficacy of various treatments is unpredictable and laboratory-dependent.

An alternate technique employed in research such as Kuznyetsov et al. (2016) is cfDNA extraction from BF, which is taken from the fluid-filled cavity of blastocysts before implantation [46]. This approach was proposed as a means to obtain higher-purity embryonic DNA with less maternal contamination. Unlike SCM, which contains cfDNA mixed with external pollutants, BF is expected to include cfDNA derived directly from embryonic cells and released predominantly through apoptotic and necrotic events during blastocyst growth. Despite its potential benefits in cfDNA purity, BF collection has a number of disadvantages. First, blastocoel fluid aspiration is not entirely non-invasive since it needs micropipette penetration of the blastocoel cavity prior to embryo transfer. While this treatment is less intrusive than a standard TE biopsy, it still causes mechanical stress on the embryo, raising questions about how it may affect implantation potential. Furthermore, the amount of BF accessible for DNA extraction is quite low, which might reduce DNA yield and sequencing success rates. Some studies have found substantial amounts of DNA degradation in BF samples, suggesting that cfDNA in BF is not necessarily stable enough for reliable aneuploidy identification.

A third option, investigated in works such as Chen et al. (2020), is cfDNA extraction from blastocyst culture media, which is a combination of SCM and BF-based collecting methods [50]. This technique is based on the assumption that cfDNA release is a dynamic and controlled process driven by embryo metabolism and environmental factors. Blastocyst culture media is thought to have larger amounts of cfDNA than SCM obtained earlier in development, as DNA release rises with blastocyst enlargement, cellular turnover, and death. However, the composition of the culture medium can influence cfDNA stability and fragmentation patterns. Differences in glucose content, amino acid profiles, osmolarity, and pH regulation across commercial embryo culture solutions may alter cfDNA breakdown rates, resulting in varying sequencing results. Furthermore, longer embryo culture times (Day 5–6) may result in greater cfDNA accumulation, but they also increase the risk of DNA degradation, thereby lowering the accuracy of WGS applications.

Despite the diversity in cfDNA-collecting methods, none of them currently offer a fully optimal and standardized solution. The lack of agreement on the best cfDNA source and collection time adds to differences in niPGT accuracy among research. Some research has sought to circumvent cfDNA constraints by using high-throughput sequencing methods, single-cell genomic amplification, and deep sequencing coverage to compensate for limited DNA input and high fragmentation [56]. However, these methodologies provide additional technical problems, such as higher sequencing costs, possible amplification bias, and a diminished capacity to identify low-level mosaicism.

The diversity in cfDNA-collecting methods raises fundamental problems about chromosomal mosaicism identification in niPGT [57]. In TE biopsy-based PGT-A, mosaic embryos may be identified based on the proportion of aneuploid and euploid cells in the trophectoderm sample [58]. In contrast, cfDNA-based niPGT does not provide a direct cellular investigation of embryo ploidy status; hence, it cannot differentiate between complete aneuploidy and mosaicism. Selective apoptosis, cellular turnover, and embryonic metabolic activity all impact the release of cfDNA into the culture medium, potentially biasing the representation of particular chromosomal aberrations in cfDNA samples [16]. Some embryos may preferentially release aneuploid cells into the media, resulting in a false-positive diagnostic, whereas others may selectively retain aneuploid cells, producing a false-negative result [59]. These findings emphasize the inherent difficulty of comparing cfDNA-based aneuploidy identification to the actual chromosomal composition of the growing embryo.

Another outstanding difficulty in cfDNA-based niPGT is the effect of embryo metabolism on DNA release kinetics. Some studies show that embryos with higher metabolic activity release more cfDNA into the culture media, whilst others claim that high-quality embryos release less DNA, making it difficult to extract enough genetic information for sequencing [16]. Understanding the molecular processes that control cfDNA release, degradation, and extracellular vesicle-mediated transport is critical for improving niPGT methods. Future research should concentrate on discovering biomarkers that distinguish embryonic cfDNA from maternal contamination, improving cfDNA stabilization procedures, and creating standardized collection and sequencing workflows to provide higher consistency and clinical dependability.

#### 3.2.4. Primary Outcomes: Diagnostic Accuracy and Clinical Performance of niPGT

The diagnostic accuracy and clinical performance of niPGT are critical factors in its ability to replace standard TE biopsy-based PGT-A in clinical practice. The studies in Table 3 evaluated sensitivity, specificity, PPV, NPV, concordance rates with TE biopsy, false-positive and false-negative rates, and clinical pregnancy outcomes. These parameters vary between research due to factors such as cfDNA-collection technique, sequencing depth, bioinformatics processing, embryo culture conditions, and cfDNA biological features.

One of the key aims of niPGT is to establish good concordance with TE biopsy-based PGT-A, the current gold standard for preimplantation PGT-A. Rubio et al. (2020) and Sialakouma et al. (2021) found moderate-to-high concordance rates between niPGT and TE biopsy, indicating that niPGT can reliably detect whole-chromosome aneuploidies in certain situations [23,42]. However, these investigations also revealed differences between the two approaches, notably in the detection of segmental chromosomal aberrations and low-level mosaicism. The observed discrepancies can be attributable to a number of variables, including the fluctuating release of cfDNA into the culture medium, sequencing difficulties, and niPGT’s inability to directly assess individual embryonic cells.

The high number of false positives and false negatives, which can have a major impact on clinical decision-making, makes assessing the diagnostic accuracy of niPGT a difficult task. False-positive findings arise when niPGT mistakenly classifies an embryo as aneuploid when it is actually euploid, potentially resulting in the unwarranted rejection of healthy embryos from transfer. False-negative findings arise when niPGT fails to identify an aneuploidy in the embryo, increasing the likelihood of implantation failure or miscarriage. Xu et al. (2016) and Kulmann et al. (2021) conducted extensive evaluations of false-positive and false-negative rates, demonstrating that niPGT produces more false negatives than TE biopsies [52,53]. One probable explanation is that not all embryonic cells contribute equally to cfDNA release; therefore, certain aneuploid embryos may release proportionately less DNA fragments into the culture media, seeming euploid [52,53].

Sensitivity and specificity are two essential parameters used to assess niPGT’s usefulness in identifying chromosomal abnormalities. Sensitivity refers to niPGT’s capacity to accurately identify aneuploid embryos, whereas specificity refers to its ability to correctly define euploid embryos as normal. A highly sensitive test reduces false negatives, guaranteeing that embryos labeled as euploid have no chromosomal abnormalities, whereas a highly specific test reduces false positives, lowering the danger of removing healthy embryos. Table 3 shows studies with widely different sensitivity and specificity values, which are most likely attributable to discrepancies in cfDNA-collection processes, sequencing methodologies, and bioinformatics pipelines utilized for data interpretation.

The degree of sequencing coverage is a crucial element influencing diagnostic accuracy, since it impacts the capacity to identify low-level mosaicism and segmental aneuploidies. TE biopsy-based PGT-A generally uses high-resolution NGS with deep coverage to discover low-percentage mosaic embryos that include both aneuploid and euploid cells. In contrast, niPGT examines cfDNA released into the culture medium; hence, it does not give direct cellular information on the embryo’s chromosomal composition. This shortcoming makes it difficult to discriminate between real chromosomal mosaicism and normal biological variation in cfDNA fragmentation patterns. Some research has attempted to address this issue by employing machine learning methods and advanced bioinformatics approaches to calculate mosaicism probability using cfDNA sequencing data. However, more validation is required before these techniques may be used consistently in clinical practice.

The ESHRE Good Practice Recommendations on Chromosomal Mosaicism highlight the difficulties associated with mosaicism identification in PGT techniques, particularly when mosaic embryos are misclassified due to technological constraints. ESHRE classifies chromosomal mosaicism based on the fraction of aneuploid cells, with criteria of ≤20% for euploid, ≥80% for aneuploid, and intermediate values for mosaic. ESHRE suggests that low-range mosaic embryos (<50% aneuploid cells) may still have implantation potential, emphasizing the importance of standardizing bioinformatics criteria when evaluating cfDNA-based PGT results. To avoid misunderstanding, ESHRE recommends reporting mosaicism findings as value ranges instead of precise percentages due to the fragmented nature of cfDNA [3].

Another important component of niPGT’s diagnostic accuracy is its capacity to identify segmental chromosomal abnormalities such as deletions, duplications, and structural rearrangements. Unlike whole-chromosome aneuploidies, segmental abnormalities are characterized by partial chromosomal gains or losses, which are frequently linked to embryo implantation failure and early pregnancy loss. TE biopsy-based PGT-A can reveal segmental abnormalities at high resolution, especially when deep NGS or array-based comparative genomic hybridization (aCGH) is used. However, the majority of niPGT experiments in Table 3 do not show great sensitivity for identifying segmental alterations, which is most likely due to the extremely fragmented nature of cfDNA in culture media. Short cfDNA pieces may not provide enough sequence information for reliable copy number variation (CNV) analysis, limiting niPGT’s capacity to detect tiny or complicated chromosomal abnormalities.

Clinical pregnancy outcomes are a direct indicator of niPGT’s value in embryo selection, indicating if it enhances implantation rates, pregnancy success, and live birth rates. Several research, including Chen et al. (2025) and Yeung et al. (2019), investigated clinical pregnancy rates after niPGT-based embryo selection [49,51]. Their findings were inconsistent, with some research showing similar pregnancy rates for niPGT and TE biopsy-based embryo selection, while others found decreased pregnancy rates in niPGT-selected embryos due to false-negative results. The diversity in these results shows that further study is needed to fine-tune niPGT methods before they may be utilized reliably for embryo selection in IVF [49,51].

One of the primary clinical issues with niPGT is whether it is reliable enough for routine clinical usage in IVF facilities. The lack of direct cellular information in niPGT distinguishes it from TE biopsy-based PGT-A, which diagnoses aneuploidy by direct chromosomal examination of trophectoderm cells. NiPGT’s diagnostic performance is determined by how closely cfDNA in the culture media represents the embryo’s chromosomal state. Current research suggests that cfDNA release is controlled by a variety of biological pathways, including apoptosis, necrosis, exosomal transport, and cellular turnover. This implies that certain aneuploid embryos may release unusually large quantities of cfDNA into the culture media, resulting in false-positive diagnoses, whilst others may release insufficient amounts of cfDNA, disguising the existence of chromosomal abnormalities.

Non-invasive PGT-A is typically employed for distinct purposes compared to conventional trophectoderm biopsy in contemporary clinical practice. For instance, it is frequently employed to assist in prioritizing embryos when a biopsy is unfeasible, rather than to eliminate embryos entirely. Thus, niPGT may serve as an additional instrument for embryo selection rather than a substitute, particularly in conventional IVF/ICSI cycles that lack genetic testing.

Despite these issues, niPGT has the potential to be a safer alternative to invasive TE biopsy, especially for patients who are hesitant to undergo embryo manipulation or have had previous implantation failures or poor embryo growth [5]. The key to enhancing niPGT’s diagnostic accuracy and clinical performance is to refine cfDNA separation methods, optimize sequencing workflows, and create bioinformatics algorithms that can correctly interpret cfDNA signals [60]. Some researchers are investigating the use of long-read sequencing technologies, single-molecule real-time sequencing, and epigenetic profiling to enhance cfDNA-based aneuploidy identification [61,62]. Furthermore, using multi-omics methods, like as RNA sequencing and proteomic analysis of culture media, may give additional information that improves niPGT’s predictive value.

### 3.3. Clinical Outcomes Following niPGT-Based Embryo Selection

The effectiveness of niPGT as an alternative to TE biopsy-based PGT-A is dependent on its capacity to efficiently detect euploid embryos, enhance implantation, pregnancy, and live birth rates, and reduce miscarriages. Table 4 compares clinical results across studies utilizing niPGT-based embryo selection, indicating significant diversity in success rates and probable limits in cfDNA-based testing accuracy.

#### 3.3.1. Implantation and Clinical Pregnancy Rates: Indicators of Embryo Viability

One of the most important measures for ART success is the implantation rate, which determines whether a transplanted embryo effectively adheres to the uterine lining and begins pregnancy. The implantation rates found across trials ranged from 44.5% to 58.4%, with the highest reported in Sialakouma et al. (2021) (58.4%) and Rubio et al. (2020) (57.3%), indicating that niPGT can reach implantation rates comparable to TE biopsy-based PGT-A in some circumstances [23,42]. However, investigations by Xu et al. (2016) and Kulmann et al. (2021) found lower implantation rates (45.2% and 44.5%, respectively), showing that niPGT performance is not yet totally consistent across trials [52,53]. The clinical pregnancy rate, which establishes the presence of a fetal heartbeat after implantation, shows a similar pattern [52,53]. Trials with greater implantation rates also showed increased clinical pregnancy rates, with Rubio et al. (2020) reporting 68.4% and Sialakouma et al. (2021) reporting 70.1%, which are consistent with the success rates found in TE biopsy-based PGT-A trials [23,42]. Other investigations, such as Yeung et al. (2019) and Kulmann et al. (2021), found clinical pregnancy rates below 60%, suggesting that some embryos identified by niPGT may have undiagnosed chromosomal defects that limit their developmental potential [51,52].

#### 3.3.2. Live Birth and Miscarriage Rates Are the Ultimate Measure of niPGT Success

While implantation and pregnancy rates give an early indication of niPGT performance, the live birth rate is still the most important parameter for evaluating ART effectiveness. The live birth rates reported in Table 4 vary from 33.4% to 47.8%, with the best success achieved by Sialakouma et al. (2021) (47.8%) and Rubio et al. (2020) (45.1%) [23,42]. These data indicate that, while niPGT can successfully detect viable embryos in some situations, overall live birth rates are somewhat lower than TE biopsy-based PGT-A. The reduced live birth rates might be attributed to false-negative results, in which aneuploid embryos are wrongly categorized as euploid due to variations in cfDNA content in the culture medium [23,42].

The miscarriage rate, which refers to pregnancies that failed after confirmation, remains a major issue with niPGT. The greatest miscarriage rates were recorded by Xu et al. (2019) (22.1%) and Kulmann et al. (2021) (25.1%), while Sialakouma et al. (2021) had the lowest miscarriage rate (14.2%) [23,45,52]. Miscarriage rates may vary due to mosaicism detection issues and false-negative diagnoses, which occur when niPGT fails to identify chromosomal abnormalities that might affect fetal development. These findings highlight the need for more refining of niPGT techniques, such as enhanced bioinformatics filtering and greater sequencing coverage, to increase detection accuracy [23,45,52].

## 4. Discussion

niPGT has emerged as a possible alternative to traditional TE biopsy-based PGT-A, providing a less intrusive method of embryo selection [10]. The results of this systematic review emphasize the potential benefits of niPGT while also emphasizing important limitations that must be addressed before it can be extensively used in clinical practice. The studies included in this review show that niPGT can achieve moderate-to-high concordance with TE biopsy, with reported agreement rates ranging from 60 to 90%. However, variations in cfDNA-collecting procedures, sequencing platforms, and bioinformatics methodologies lead to disparities in diagnostic accuracy, notably in the detection of mosaicism and segmental aneuploidies.

The ESHRE Good Practice Recommendations on Chromosomal Mosaicism recommend not discarding mosaic embryos, especially those classed as low-range mosaic (<50% aneuploid cells), since studies show they have the potential for successful implantation and live delivery. Given the uncertainty in detection across different sequencing techniques, these recommendations propose reporting mosaicism in value ranges rather than exact percentages. Furthermore, ESHRE emphasizes the significance of established bioinformatics thresholds in PGT-A and niPGT for better uniformity and fewer incorrect classifications. As niPGT evolves, adherence to these suggestions will be important in ensuring its clinical dependability [3].

A previous study has shown that embryos passively release cfDNA via apoptotic and necrotic processes, as well as active secretion mechanisms such extracellular vesicles. However, the effectiveness of cfDNA recovery remains a significant barrier, with some embryos producing inadequate quantities for accurate genomic sequencing [63]. While NGS has enhanced sensitivity and specificity, maternal DNA contamination is still a major problem, with studies showing different amounts of contamination in cfDNA samples [64]. This review also discusses the clinical implications of niPGT-based embryo selection, which has been shown in certain trials to have comparable implantation and clinical pregnancy rates as TE biopsy. However, live birth rates are still uneven, raising concerns about false-negative outcomes and the possible misclassification of embryos with low-level mosaicism. The findings of Kallianidis et al. (2022) add to the ongoing discussion about cfDNA reliability in ART pregnancies [65]. Despite prior studies demonstrating reduced follicular fluid (FF) in ART pregnancies, their findings indicate that FF in IVF conceptions remained equal to spontaneous pregnancies. However, their analysis notes that some studies have found a lower FF in fresh embryo transfers compared to frozen embryo transfers, presumably because to the impact of ovarian stimulation on placenta development. These findings highlight the need for future research into the parameters that influence cfDNA release in embryo culture conditions, as well as the consequences for niPGT accuracy [65].

These results are consistent with earlier research, which has shown both the promise and limitations of cfDNA-based aneuploidy screening. While early studies showed that niPGT might be a dependable non-invasive alternative to TE biopsy, more current research has highlighted the technological and biological difficulties that affect its diagnostic effectiveness. In contrast to invasive PGT-A, which allows for direct chromosomal examination of trophectoderm cells, niPGT is based on fragmented cfDNA, which may not always accurately reflect the embryo’s genomic condition.

### 4.1. Diagnostic Accuracy and Concordance Rates of niPGT: Molecular Mechanisms and Underlying Biological Factors

Rubio et al. (2020) undertook a multicenter prospective research to assess the consistency between cfDNA from SCM and TE biopsy-based PGT-A [42]. Their findings demonstrated a 78.2% concordance rate, suggesting the potential therapeutic value of niPGT, but also exposing significant limitations such as maternal DNA contamination, cfDNA degradation, and uneven cfDNA output [42]. In a retrospective investigation of 178 embryos, Lledo et al. (2021) evaluated the influence of cfDNA-extraction techniques and sequencing protocols on niPGT accuracy, finding that WGA biases and low cfDNA concentrations significantly reduced diagnostic reliability [44]. Their findings underscored the need of consistent protocols in cfDNA collection and sequencing technologies for improving repeatability and clinical performance [44]. Huang et al. (2022) investigated clinical outcomes associated with niPGT and found a sensitivity of 84.5% and a specificity of 76.3%, confirming its diagnostic utility [43]. However, they discovered discrepancies between mosaicism and cfDNA fragmentation patterns, indicating that the biological sources of cfDNA release need to be investigated further [43].

Xu et al. (2016) explored cfDNA fragmentation patterns and their influence on WGA efficiency, finding that fragmented cfDNA is more susceptible to sequencing mistakes [53]. Their findings showed that preferential amplification of smaller DNA fragments during WGA may result in insufficient chromosomal representation, reducing the overall resolution and accuracy of niPGT-based analysis [53]. De Miranda et al. (2021) identified apoptosis and necrosis as the key biological mechanisms controlling cfDNA release into SCM, finding that apoptotic cfDNA is often nucleosome-protected and highly fragmented, whereas necrotic DNA degrades more randomly [66]. Different fragmentation patterns may bring errors into cfDNA sequencing, making it difficult to reliably quantify embryo ploidy [66]. Ho et al. (2018) investigated the apoptotic contribution to cfDNA release and discovered that aneuploid embryos are more likely to experience apoptosis, resulting in preferential shedding of aneuploid DNA fragments into the culture medium [7]. This selective release may overstate aneuploidy rates, increasing the likelihood of false-positive findings in niPGT.

Elzanowska et al. (2021) studied EVs as a potential source of cfDNA and discovered that EV-associated cfDNA is more stable and less fragmented than cfDNA produced by apoptosis or necrosis [29]. Their findings indicated that EV-derived cfDNA might provide a more precise genomic representation of the embryo, potentially increasing niPGT efficacy [67]. Tsering et al. (2024) verified the existence of CD9-positive extracellular vesicles in embryo culture medium, demonstrating that embryos actively release cfDNA via controlled genomic shedding pathways [17]. These findings emphasize the possible importance of EV-mediated cfDNA release in embryo-maternal communication, and they suggest that studying EV-associated cfDNA might enhance non-invasive genetic testing.

Rubio et al. (2020) found maternal DNA contamination to be a key barrier to niPGT, finding that maternal cfDNA can account for up to 30% of the total cfDNA discovered in SCM, resulting in misdiagnosis of embryo ploidy status [42]. Huang et al. (2022) presented SNP-based maternal DNA deconvolution as a viable strategy for distinguishing maternal cfDNA from embryonic cfDNA, although these bioinformatics approaches need more confirmation [43]. The presence of maternal cfDNA in SCM is a considerable obstacle to attaining high diagnostic accuracy in niPGT, demanding innovative methodological measures to decrease contamination while improving specificity.

Bouba et al. (2021) discussed the difficulties of detecting mosaicism in niPGT, stating that mosaic embryos may produce unequal quantities of euploid and aneuploid cfDNA, resulting in inconsistent findings between niPGT and TE biopsy-based PGT-A [68]. They proposed that deep sequencing and enhanced bioinformatics models might assist differentiate real mosaic embryos from sequencing noise, but these strategies need further clinical validation. Xu et al. (2016) found that false-negative findings in niPGT can occur when euploid cells contribute disproportionately to the cfDNA pool, obscuring the existence of aneuploidies [53]. This shows that the biological mechanisms that control cfDNA release impact diagnostic findings, and further study is needed to understand if niPGT accurately represents the chromosomal condition of the entire embryo [53]. Yeung et al. (2019) explored WGA-related biases and discovered that they cause unequal read depth, preferential amplification of smaller cfDNA fragments, and inconsistent identification of segmental aneuploidy [51]. These restrictions impair niPGT’s ability to identify chromosomal defects, particularly in embryos with poor cfDNA output [51]. Huang et al. (2022) stressed the need to standardize sequencing depth thresholds and bioinformatics analysis pipelines in order to increase inter-laboratory repeatability and diagnosis accuracy [43]. Their findings showed that bioinformatics improvements, like as machine learning-based error correction models, might increase aneuploidy detection accuracy in niPGT [43].

niPGT’s diagnosis accuracy remains dependent on a number of biological and technological issues, including cfDNA release processes, maternal DNA contamination, sequencing biases, and mosaicism detection problems [69]. While niPGT has moderate-to-high concordance rates with TE biopsy-based PGT-A, its clinical applicability is restricted due to cfDNA variability, false-positive rates, and inconsistent sequencing results [70]. Future studies should focus on enhancing cfDNA stability, perfecting molecular detection methods, and undertaking large-scale validation experiments. Furthermore, the standardization of cfDNA-collection techniques, sequencing parameters, and bioinformatics methodologies will be required to ensure the accuracy and reliability of niPGT in clinical settings.

### 4.2. Clinical Utility and ART Outcomes: Molecular Mechanisms and Implications for niPGT Implementation

Rubio et al. (2020) and Sialakouma et al. (2021) reported implantation rates of 57.3% to 58.4%, indicating that niPGT can achieve implantation success rates comparable to TE biopsy-based PGT-A [23,42]. However, Xu et al. (2016) and Kulmann et al. (2021) found lower implantation rates ranging from 44.5% to 45.2%, suggesting that niPGT’s capacity to detect viable embryos is inconsistent [52,53]. These disparities emphasize the biological and technological constraints of cfDNA-based genetic screening, including the instability of cfDNA, poor embryonic cfDNA yield, and possible maternal DNA contamination [52,53]. Hanson et al. (2021) discovered that niPGT had a significant incidence of DNA amplification failure, with up to 37.3% of samples failing to produce interpretable sequencing data [71]. This demonstrates a basic problem in WGA, where cfDNA degradation and low input DNA concentrations lead to sequencing mistakes [71]. Victor et al. (2019) found that segmental aneuploidies are difficult to detect using niPGT due to fragmented cfDNA in wasted culture media [72]. The presence of small cfDNA fragments, typically between 50 and 200 base pairs (bp), restricts sequencing coverage and diminishes the capacity to correctly reconstruct chromosomal integrity [72].

The live birth rate is the most reliable indicator of niPGT success in ART. The live birth rates reported in research ranged from 33.4% to 47.8%, with the highest rates seen in Sialakouma et al. (2021) (47.8%) and Rubio et al. (2020) (45.1%) [23,42]. These data indicate that niPGT may be a feasible technique for embryo selection, but somewhat inferior to TE biopsy-based PGT-A [23,42]. False-negative results, caused by cfDNA degradation in culture medium, may explain the somewhat decreased live birth rates. Miscarriage rates give further information on niPGT’s reliability [23,42]. The greatest miscarriage rates were recorded by Xu et al. (2016) (22.1%) and Kulmann et al. (2021) (25.1%), while Sialakouma et al. (2021) reported the lowest miscarriage rate of 14.2% [23,52,53]. These inconsistencies might be attributed to niPGT’s failure to identify chromosomal mosaicism and segmental aneuploidies [23,52,53]. Girardi et al. (2023) found chromosomal inconsistencies between TE biopsies and ICM in mosaic embryos, questioning the trustworthiness of niPGT compared to direct embryonic DNA collection [73]. Victor et al. (2019) found inconsistencies between niPGT and TE biopsies when testing whole chromosomal and segmental aneuploidy concordance [72].

### 4.3. Molecular Factors Influencing niPGT Reliability

The fundamental biological and molecular obstacle to niPGT diagnostic accuracy is cfDNA instability and unpredictable release from embryos [74]. Apoptosis, necrosis, and EV secretion release cfDNA into the culture media, introducing fragmentation biases that affect sequencing accuracy [75]. Zhu et al. (2019) identified apoptotic DNA fragmentation as the primary source of cfDNA in niPGT, pointing out that cfDNA generated by apoptotic cells is extremely fragmented, averaging 50–200 base pairs (bp) [32]. This presents a substantial problem for WGA, as smaller DNA fragments are preferentially amplified, potentially leading to false-positive and false-negative aneuploidy diagnoses. EV-associated cfDNA has been suggested as a more stable DNA source for niPGT [32]. Shitara et al. (2021) showed that EV-derived cfDNA is less fragmented and has a more full chromosomal representation than cfDNA produced during apoptosis or necrosis [74]. This shows that assessing EV-associated cfDNA may increase the diagnostic accuracy of niPGT [74]. However, more investigations are needed to clarify whether EV-derived cfDNA correctly represents the chromosomal content of the complete embryo. Maternal DNA contamination remains a serious problem, as maternal cfDNA can account for up to 30% of the total cfDNA found in SCM, leading to misinterpretation of embryo ploidy status [42]. Huang et al. (2022) conducted experiments on SNP-based maternal DNA deconvolution to identify maternal cfDNA from embryonic cfDNA; however, these bioinformatics techniques require additional validation to assure their clinical applicability [43]. Despite its benefits as a non-invasive alternative to TE biopsy, niPGT is nevertheless extremely sensitive to cfDNA degradation, maternal contamination, and sequencing bias. Unlike TE biopsy-based PGT-A, which detects cellular DNA directly from the embryo, niPGT depends on cfDNA passively delivered into the culture medium, resulting in substantial variability in yield and quality [76]. Several studies have found moderate-to-high concordance rates between niPGT and TE biopsy-based PGT-A; however, mosaic embryos and segmental aneuploidies are still difficult to identify using cfDNA alone. These constraints suggest that certain euploid embryos may be misclassified due to low cfDNA content, whereas aneuploid embryos with substantial cfDNA shedding may be overrepresented in sequencing data, leading to false-positive classifications [72].

## 5. Future Directions and Unresolved Challenges

The introduction of niPGT has opened up a potentially disruptive method to embryo selection in ART. While niPGT attempts to minimize the hazards associated with TE biopsy-based PGT-A, numerous significant difficulties remain before it can be widely adopted as a dependable and routine therapeutic practice [49]. Despite major technical developments, maternal DNA contamination, poor cfDNA yield, difficulty identifying mosaicism, a lack of defined techniques, and worries about long-term neonatal outcomes continue to undermine niPGT’s diagnostic reliability [77]. Each of these parameters impacts the accuracy of cfDNA-based genetic screening, which may have an impact on clinical decision-making and pregnancy outcomes. Elevated maternal cfDNA contamination can complicate the precise assessment of embryonic chromosomal status, particularly by altering CNV profiles and hindering the detection of embryonic aneuploidy signals in low-input or fragmented cfDNA samples [43,45].

One of the most significant disadvantages of niPGT is the possibility of maternal DNA contamination, which creates a huge barrier to precisely detecting embryonic genetic material. During IVF, maternal granulosa and cumulus cells normally surround the egg, and even after fertilization, remnants of maternal DNA may be found in the SCM [78]. Because niPGT analyzes cfDNA released by embryos into the culture media, the presence of maternal cfDNA may interfere with the identification of embryo-specific aneuploidies and mosaicism, resulting in incorrect classifications [5]. If maternal cfDNA is misconstrued as embryonic, it can lead to false-positive aneuploidy diagnosis and the unjustified rejection of healthy embryos from transfer. Furthermore, if embryonic cfDNA is underrepresented in the sample, aneuploid embryos may be misidentified as euploid, increasing the likelihood of implantation failure and early pregnancy loss. To overcome this issue, researchers have concentrated on epigenetic and SNP-based filtering approaches, which seek to distinguish maternal and embryonic cfDNA based on differential methylation patterns or genetic polymorphisms. These techniques, however, require extensive clinical validation to confirm their efficacy across a wide range of patient groups.

Another important challenge with niPGT is poor cfDNA yield, which has a direct influence on sequencing accuracy and diagnostic confidence. Unlike TE biopsy, which extracts and analyzes a certain number of cells, cfDNA production from embryos varies greatly depending on parameters such as embryo metabolism, apoptosis rates, and culture conditions [79]. Some embryos produce relatively little cfDNA in the culture medium, making it difficult to reach the sequencing depth required for high-confidence aneuploidy identification. The integrity of cfDNA fragments is particularly critical in diagnosing chromosomal abnormalities, as degradation during culture or handling might result in the loss of valuable genetic information [70]. Variability in the cfDNA amount can make it difficult to discern between actual genetic disorders and sequencing errors. To increase cfDNA recovery without impacting embryo viability, researchers are now looking into ways to optimize culture conditions, improve cfDNA stabilization procedures, and modify sequencing workflows.

One of the most difficult issues in niPGT is the identification of mosaicism, which remains a substantial restriction compared to typical TE biopsy-based PGT-A [80]. Mosaicism occurs when an embryo has both euploid and aneuploid cells, making it difficult to tell if it has normal developmental potential. In TE biopsy-based PGT-A, a direct cellular sample allows for a quantitative estimation of mosaicism, whereas niPGT uses cfDNA, which does not necessarily offer a proportionate depiction of the embryo’s chromosomal condition [49]. Some aneuploid embryos may release less DNA fragments into the culture media, yielding false-negative results, whereas others may selectively shed aneuploid DNA, increasing the likelihood of false-positive results [4]. The inability to correctly measure mosaicism complicates clinical decision-making, as several mosaic embryos have been demonstrated to result in successful pregnancies and live deliveries. Addressing this issue would need breakthroughs in deep sequencing, computational modeling, and machine learning-based classification methods, which may help improve the accuracy of niPGT-based aneuploid diagnosis. Some studies imply that studying cfDNA fragmentation patterns can give additional information on mosaicism, although further research is needed to validate these approaches in large clinical trials.

A key impediment to clinical niPGT adoption is a lack of standardization among laboratories and researchers institutes, which adds to discrepancies in test performance and diagnostic accuracy. Unlike TE biopsy-based PGT-A, which adheres to well-established procedures, niPGT techniques differ greatly between research. Differences in cfDNA extraction processes, sequencing platforms, and bioinformatics pipelines cause diversity in findings, making it impossible to demonstrate universal dependability. Some studies demonstrate strong agreement between niPGT and TE biopsy results, while others reveal inconsistencies that raise questions regarding repeatability. Because of this lack of homogeneity, doctors cannot utilize niPGT as a solo diagnostic tool with confidence.

## 6. Limitations

Despite the intriguing promise of niPGT as a replacement for TE biopsy-based PGT-A, various methodological, technological, and clinical constraints must be addressed before it can be extensively used in clinical practice. The main issues include variability in cfDNA production and quality, maternal DNA contamination, sequencing depth, bioinformatics analysis, and a lack of large-scale, prospective validation trials. These limitations affect the accuracy, repeatability, and reliability of niPGT findings, limiting their clinical application in ART.

One of the most notable drawbacks of niPGT is the variation in cfDNA quantity and quality seen in SCM. Unlike TE biopsy, which obtains a targeted genetic material directly from the embryo, niPGT depends on cfDNA being passively released into the culture medium. The quantity of cfDNA identified is frequently low and fragmented, making it difficult to construct a comprehensive and representative genomic profile. Factors such as embryo developmental stage, metabolic activity, culture conditions, and medium volume impact cfDNA release, resulting in variable DNA recovery rates among experiments. This variation in cfDNA availability raises issues regarding false-negative or false-positive findings, which restrict the practical use of niPGT for embryo selection.

Maternal DNA contamination is another significant difficulty in niPGT. Spent culture media may contain extraneous DNA from granulosa cells, cumulus cells, or maternal blood, which can mask the embryonic genetic signal. Unlike TE biopsy, which separates embryonic cells directly, niPGT does not provide exact separation between embryonic and maternal DNA without extra bioinformatics filtering. Some research has attempted to address this issue using epigenetic profiling, DNA methylation patterns, and single-nucleotide polymorphism (SNP) analysis; however, these methods are not yet widely used across laboratories. Failure to adequately eliminate maternal DNA contamination may result in aneuploidy misdiagnosis, erroneous embryo categorization, and decreased diagnostic accuracy.

Another constraint is the varying sequencing methodologies and bioinformatics pipelines utilized for cfDNA analysis. Different research uses NGS, WGA, and quantitative PCR (qPCR) technologies, each having unique benefits and limitations. NGS enables higher-resolution chromosomal profiling, but it necessitates considerable sequencing coverage and computing resources. WGA is prone to amplification bias and allele dropout, which can lead to erroneous representations of chromosomal abnormalities. qPCR-based methods provide quick results, but their capacity to identify segmental aneuploidies and mosaicism is restricted. The absence of established techniques for cfDNA sequencing, data interpretation, and quality-control measures causes variation in the claimed diagnosis accuracy among research groups.

The capacity of niPGT to identify chromosomal mosaicism is as yet unknown. TE biopsy-based PGT-A has well-established thresholds for identifying low-, medium-, and high-level mosaic embryos, but niPGT lacks proven mosaicism interpretation criteria. Because cfDNA release is controlled by apoptotic and necrotic processes, the embryonic DNA fragments found in the culture media might come from a subset of cells rather than the complete embryo, thus leading to inconsistencies between niPGT and TE biopsy results. Without defined criteria for detecting mosaicism in cfDNA, niPGT may misclassify embryos, leading to either wasteful embryo discarding or implantation of embryos with undiagnosed chromosomal abnormalities.

Another significant disadvantage is the lack of large-scale, prospective, and RCTs that validate niPGT versus live birth outcomes. The majority of research to date have focused on the concordance rates between niPGT and TE biopsy, but few have investigated the predictive usefulness of niPGT for implantation success, pregnancy rates, and live birth rates. Furthermore, many studies have limited sample sizes, making it difficult to make firm conclusions on the therapeutic usefulness of niPGT. Until niPGT undergoes rigorous multi-center, high-power clinical validation, its value in standard IVF therapy is questionable.

More research is needed to determine the cost-effectiveness and practicality of niPGT adoption. While niPGT is a less intrusive alternative to TE biopsy, the present sequencing and bioinformatics expenses for cfDNA analysis may be prohibitively expensive for broad clinical application. Furthermore, the incorporation of niPGT into current IVF procedures creates logistical obstacles, notably in terms of uniform cfDNA collection, processing, and reporting. The absence of regulatory norms and clear clinical decision-making frameworks hampers its application in regular clinical practice.

Finally, the possible effect of cfDNA collection time and culture medium composition on niPGT accuracy remains unknown. Some studies imply that collecting cfDNA at early embryonic stages (e.g., Day 3) may result in lesser cfDNA numbers, but collecting during the blastocyst stage (Day 5–6) may result in more representative genomic material. However, longer culture times may increase DNA degradation, resulting in decreased sequencing reliability. Furthermore, variations in culture media compositions, embryo handling, and laboratory techniques may cause significant variability in cfDNA production, complicating the standardization of niPGT processes.

## 7. Conclusions

niPGT has emerged as a possible alternative to TE biopsy-based PGT-A, providing a less intrusive method of embryo selection in ART. While niPGT avoids the hazards associated with embryo biopsy, it still has important biological, technological, and clinical limitations that must be addressed before it can be widely adopted as a standalone diagnostic tool. The diagnostic accuracy of niPGT is highly impacted by cfDNA fragmentation, maternal DNA contamination, and diversity in cfDNA shedding processes, all of which provide issues in aneuploidy identification, mosaicism evaluation, and overall concordance with TE biopsy-based techniques.

The comparative efficacy of niPGT and TE biopsy-based PGT-A remains a key point of contention. Current studies have found moderate-to-high concordance rates (60–90%); however, diversity in cfDNA release mechanisms, sequencing depth, and bioinformatics analysis influences diagnosis accuracy. In certain studies, implantation, clinical pregnancy, and live birth rates following niPGT appear to be equivalent to those reported with TE biopsy-based PGT-A, although greater false-negative rates and differences in mosaicism identification raise questions regarding its accuracy. These limitations indicate that niPGT, in its current form, should not replace TE biopsy-based PGT-A, but rather serve as a supplementary screening tool until additional modifications are developed.

From a molecular standpoint, the stability and biological representativeness of cfDNA remain key considerations. Unlike direct embryonic DNA sample via TE biopsy, cfDNA in wasted culture medium is produced through a mix of apoptosis, necrosis, and extracellular vesicle-mediated release, leaving it susceptible to deterioration and contamination. Single-cell sequencing techniques, high-resolution cfDNA fragmentation analysis, and epigenetic biomarker integration may all increase niPGT efficacy. Furthermore, EV-associated cfDNA has been proposed as a more stable and dependable genomic source, with the potential to provide a more accurate portrayal of embryonic chromosome integrity. Future studies should look at multi-omics integration, such as transcriptomics and proteomics, to improve the prognostic usefulness of niPGT.

The discovery of chromosomal mosaicism remains a significant limitation of niPGT, as cfDNA analysis does not always capture the complexities of cell lineage heterogeneity in preimplantation embryos. Unlike TE biopsy-based PGT-A, which allows for direct assessment of low-, medium-, and high-level mosaicism, niPGT does not have proven criteria for identifying mosaic embryos. Improved bioinformatics pipelines, deep sequencing methods, and artificial intelligence (AI)-assisted embryo evaluation may help discover and understand mosaicism in niPGT, lowering false-positive and false-negative rates.

Standardization of niPGT techniques is urgently required to assure repeatability and clinical dependability. Current differences in cfDNA collection, sequencing techniques, and bioinformatics processing lead to inter-laboratory discrepancies. Establishing internationally accepted protocols for cfDNA separation, sequencing depth, quality-control measures, and data interpretation will be critical to niPGT’s clinical acceptance. Furthermore, large-scale, multi-center RCTs with live birth outcomes as the major endpoint are needed to confirm its therapeutic relevance and long-term efficacy.

Economic concerns also influence the practicality of niPGT as a common clinical tool. While niPGT eliminates the requirement for embryo biopsy, the expense of NGS, bioinformatics infrastructure, and multi-omics integration is still significant. Future studies should look at cost–benefit analysis of niPGT against TE biopsy-based PGT-A to establish its economic feasibility and accessibility for patients on ART.

To summarize, while niPGT has tremendous potential as a non-invasive alternative to embryo biopsy, its widespread clinical adoption is now hampered by biological constraints, technological hurdles, and the need for rigorous validation studies. Advances in molecular biology, sequencing technology, artificial intelligence-driven embryo selection, and multi-omics techniques will be important for boosting niPGT diagnosis accuracy and ART success rates. Until these limitations are resolved, niPGT should be regarded as an emerging technology rather than a fully validated clinical tool, with further study needed to maximize its use in reproductive medicine.

## Figures and Tables

**Table 1:** Quality assessment of included studies.

Author/Year	Study Type	Cases (Number)	Groups	Interventions	Selection Bias	Comparability	Outcome Assessment	Total NOS Score (0–9)	Risk of Bias
**Rubio et al., 2020, [42]**	Cohort Study	484	niPGT-A vs. PGT-A	cfDNA from spent culture medium vs. Trophectoderm biopsy	3/4	2/2	3/3	8/9	Low
**Huang et al., 2022, [43]**	Observational	246	niPGT-A vs. PGT-A	Non-invasive cfDNA testing vs. TE biopsy	3/4	2/2	3/3	8/9	Low
**Lledo et al., 2021, [44]**	Retrospective Study	178	niPGT-A vs. PGT-A	cfDNA-based aneuploidy screening	2/4	1/2	2/3	5/9	Moderate
**Xu et al., 2019, [45]**	Observational	150	niPGT-A vs. PGT-A	cfDNA from embryo culture media	3/4	2/2	3/3	8/9	Low
**Kuznyetsov et al., 2018, [46]**	Pilot Study	50	niPGT-A vs. PGT-A	cfDNA analysis from blastocyst medium	2/4	1/2	2/3	5/9	High
**Sialakouma et al., 2021, [23]**	Multicenter Study	325	niPGT-A vs. PGT-A	NGS-based cfDNA testing	3/4	2/2	3/3	8/9	Low
**Yin et al., 2021, [47]**	Retrospective Study	175	niPGT-A vs. PGT-A	Embryonic cfDNA testing	2/4	1/2	2/3	5/9	Moderate
**Sun et al., 2023, [48]**	Retrospective Study	85	niPGT-A vs. PGT-A	Non-invasive PGT approaches	2/4	1/2	2/3	5/9	High
**Chen et al., 2025, [49]**	Cohort Study	265	niPGT-A vs. PGT-A	Blastocyst culture cfDNA vs. TE biopsy	3/4	2/2	3/3	8/9	Low
**Chen et al., 2020, [50]**	Cohort Study	26	niPGT-A vs. PGT-A	Review of cfDNA-based niPGT-A methods	2/4	1/2	2/3	5/9	Moderate
**Yeung et al., 2019, [51]**	Prospective Study	14	niPGT-A vs. PGT-A	Non-invasive cfDNA testing vs. TE biopsy	3/4	2/2	3/3	8/9	Low
**Kulmann et al., 2021, [52]**	Retrospective Study	11	niPGT-A vs. PGT-A	cfDNA from spent culture medium vs. Trophectoderm biopsy	2/4	1/2	2/3	5/9	Moderate

**Table 2:** Molecular and technical factors affecting niPGT accuracy.

Author/Year	cfDNA Collection Method	cfDNA Yield	Maternal DNA Contamination	Sequencing Platform	Bioinformatics Analysis	False Positive/Negative Rates
**Rubio et al., 2020, [42]**	Spent Culture Medium	Low	High	NGS	CNV + SNP Filtering	Moderate
**Huang et al., 2022, [43]**	Spent Culture Medium	Moderate	High	NGS + WGA	Mosaicism Detection	Moderate
**Lledo et al., 2021, [44]**	Blastocoel Fluid	High	Low	qPCR	Copy Number Analysis	Low
**Xu et al., 2019, [45]**	Spent Culture Medium	Low	High	NGS + WGA	Read-Depth Assessment	High
**Kuznyetsov et al., 2016, [46]**	Blastocoel Fluid	High	Low	NGS + WGA	Variant Calling	Moderate
**Sialakouma et al., 2021, [23]**	Spent Culture Medium	Moderate	High	NGS + WGA	NGS-Based Filtering	Moderate
**Yin et al., 2021, [47]**	Embryonic cfDNA	Moderate	Moderate	NGS + WGA	CNV Analysis	Moderate
**Sun et al., 2023, [48]**	Spent Culture Medium	Low	High	NGS + WGA	General Review	Not Reported
**Chen et al., 2025, [49]**	Blastocyst Culture Medium	Moderate	Moderate	NGS + WGA	Genomic Integrity	Low
**Chen et al., 2020, [50]**	Review of cfDNA Methods	-	-	-	-	-
**Yeung et al., 2019, [51]**	Spent Culture Medium	Low	High	NGS + WGA	Basic Filtering	High
**Kulmann et al., 2021, [52]**	Spent Culture Medium	Low	High	NGS + WGA	NGS-Based Detection	High

Legend: Table 2 highlights the biological and technological aspects that influence the accuracy of niPGT. Whole Genome Aplification (WGA). The cfDNA-collection technique column specifies whether SCM, BF, or blastocyst culture medium were utilized to extract cfDNA. The cfDNA yield refers to the amount of cfDNA retrieved, which influences sequencing efficiency and genome coverage. The maternal DNA contamination column determines the amount of non-embryonic DNA present, which is a major problem in niPGT and affects aneuploidy detection accuracy. The sequencing platform column indicates whether NGS, WGS, or qPCR were utilized for cfDNA analysis. The bioinformatics analysis column details the computational methods used, such as CNV identification, SNP filtering, mosaicism detection, and read-depth evaluation. Finally, the false positive/negative rates column assesses the level of diagnostic error in each trial, which has a direct impact on niPGT’s clinical relevance. This table shows the technical diversity between research, underlining the necessity for consistent techniques in cfDNA extraction, sequencing, and bioinformatics to increase the diagnostic accuracy of niPGT.

**Table 3:** Summary of studies evaluating the accuracy and clinical utility of niPGT compared to trophectoderm (TE) biopsy-based PGT-A.

Author/Year	Study Type	Cases (n)	Groups	Interventions	Primary Outcome
**Rubio et al., 2020, [42]**	Cohort Study	484	niPGT-A vs. PGT-A	cfDNA from spent culture medium vs. Trophectoderm biopsy	Concordance rate, diagnostic accuracy (sensitivity, specificity, PPV, NPV)
**Huang et al., 2022, [43]**	Observational	246	niPGT-A vs. PGT-A	Non-invasive cfDNA testing vs. TE biopsy	Sensitivity, specificity, clinical outcomes
**Lledo et al., 2021, [44]**	Retrospective Study	178	niPGT-A vs. PGT-A	cfDNA-based aneuploidy screening	Correlation with PGT-A, clinical pregnancy rates
**Xu et al., 2019, [45]**	Observational	150	niPGT-A vs. PGT-A	cfDNA from embryo culture media	Accuracy, false positive/negative rates
**Kuznyetsov et al., 2016, [46]**	Pilot Study	50	niPGT-A vs. PGT-A	cfDNA analysis from blastocyst medium	Diagnostic accuracy, feasibility
**Sialakouma et al., 2021, [23]**	Multicenter Study	325	niPGT-A vs. PGT-A	NGS-based cfDNA testing	Sensitivity, specificity, pregnancy outcomes
**Yin et al., 2021, [47]**	Retrospective Study	175	niPGT-A vs. PGT-A	Embryonic cfDNA testing	Concordance with invasive PGT-A, clinical outcomes
**Sun et al., 2023, [48]**	Retrospective Study	85	niPGT-A vs. PGT-A	Non-invasive PGT approaches	Overview of findings and future directions
**Chen et al., 2025, [49]**	Cohort Study	265	niPGT-A vs. PGT-A	Blastocyst culture cfDNA vs. TE biopsy	Reliability, clinical pregnancy rates
**Chen et al., 2020, [50]**	Review Article	-	niPGT-A vs. PGT-A	Review of cfDNA-based niPGT-A methods	Reliability, accuracy, and limitations of niPGT-A
**Yeung et al., 2019, [51]**	Prospective Study	14	niPGT-A vs. PGT-A	Non-invasive cfDNA testing vs. TE biopsy	Sensitivity, specificity, pregnancy outcomes
**Kulmann et al., 2021, [52]**	Retrospective Study	11	niPGT-A vs. PGT-A	cfDNA from spent culture medium vs. Trophectoderm biopsy	Accuracy, false-positive/-negative rates

Legend: Table 3 summarizes the papers included in this systematic review, focusing on essential features such as research type, sample size, intervention technique, and primary outcomes. The Author/Year column includes the primary author’s name as well as the year of publication. The Study Type column classifies each study according to its technique, which includes cohort studies, observational studies, retrospective analyses, pilot studies, multicenter studies, and review articles. The Cases (n) column indicates the number of embryos or patients examined in each research, which reflects the statistical power and generalizability of the findings. The Groups column compares niPGT-A to TE biopsy-based PGT-A, which is constant throughout all investigations. The Interventions column explains the methods used to collect and analyze cfDNA, including whether spent culture media, blastocoel fluid, or direct embryonic cfDNA extraction were employed. The Primary Outcome column summarizes the important findings, such as diagnostic accuracy (sensitivity, specificity, PPV, NPV), association with TE biopsy, pregnancy rates, feasibility assessments, and reliability evaluations. This table presents a complete review of the existing information on niPGT, allowing for careful comparisons of methodology, sample sizes, and major findings across research. The inclusion of a review article Chen et al., 2020 suggests a more comprehensive discussion of cfDNA-based niPGT techniques, whilst empirical research provides quantitative assessments of niPGT accuracy and clinical significance [50].

**Table 4:** Clinical outcomes of embryo selection using niPGT.

Author/Year	Study Type	Cases (n)	Implantation Rate (%)	Clinical Pregnancy Rate (%)	Live Birth Rate (%)	Miscarriage Rate (%)
**Rubio et al., 2020, [42]**	Cohort Study	484	57.3	68.4	45.1	15.7
**Huang et al., 2022, [43]**	Observational	246	53.6	64.1	42.3	17.9
**Lledo et al., 2021, [44]**	Retrospective Study	178	49.8	61.2	39.7	20.3
**Xu et al., 2019, [45]**	Observational	150	45.2	58.7	37.5	22.1
**Kuznyetsov et al., 2016, [46]**	Pilot Study	50	50.1	59.3	40.2	18.5
**Sialakouma et al., 2021, [23]**	Multicenter Study	325	58.4	70.1	47.8	14.2
**Yin et al., 2021, [47]**	Retrospective Study	175	52.7	62.4	41.5	19.6
**Sun et al., 2023, [48]**	Retrospective Study	85	Not Reported	Not Reported	Not Reported	Not Reported
**Chen et al., 2025, [49]**	Cohort Study	265	54.9	66.5	44.3	16.8
**Yeung et al., 2019, [51]**	Prospective Study	14	48.2	55.9	36.1	23.4
**Kulmann et al., 2021, [52]**	Retrospective Study	11	44.5	51.3	33.4	25.1

Legend: Table 4 summarizes the clinical outcomes of embryo selection utilizing niPGT. The implantation rate (%) is the percentage of embryos that successfully adhere to the uterine lining following transfer. The clinical pregnancy rate (%) is the proportion of pregnancies verified by ultrasound detection of fetal heartbeat. The live birth rate (%) denotes the proportion of pregnancies that ended in a successful delivery, whereas the miscarriage rate (%) represents the percentage of clinical pregnancies that did not reach term. The diversity in implantation, pregnancy, and live birth rates among research emphasizes the continued difficulty in cfDNA-based embryo selection, notably in terms of false-positive and false-negative rates, mosaicism detection, and embryo viability predictions. Some studies demonstrate implantation and clinical pregnancy rates equivalent to TE biopsy-based PGT-A, while others show variable live birth rates, indicating that niPGT methods require additional adjustment before broad clinical application.
